# Distinct Functional Metagenomic Markers Predict the Responsiveness to Anti-PD-1 Therapy in Chinese Non-Small Cell Lung Cancer Patients

**DOI:** 10.3389/fonc.2022.837525

**Published:** 2022-04-21

**Authors:** Chao Fang, Wenfeng Fang, Liqin Xu, Fangfang Gao, Yong Hou, Hua Zou, Yuxiang Ma, Janne Marie Moll, Yunpeng Yang, Dan Wang, Yan Huang, Huahui Ren, Hongyun Zhao, Shishang Qin, Huanzi Zhong, Junhua Li, Sheng Liu, Huanming Yang, Jian Wang, Susanne Brix, Karsten Kristiansen, Li Zhang

**Affiliations:** ^1^ Laboratory of Genomics and Molecular Biomedicine, Department of Biology, University of Copenhagen, Copenhagen, Denmark; ^2^ BGI-Shenzhen, Shenzhen, China; ^3^ State Key Laboratory of Oncology in South China, Collaborative Innovation Center for Cancer Medicine, Sun Yat-Sen University Cancer Center, Guangzhou, China; ^4^ Department of Biotechnology and Biomedicine, Technical University of Denmark, Lyngby, Denmark; ^5^ School of Biology and Biological Engineering, South China University of Technology, Guangzhou, China; ^6^ James D. Watson Institute of Genome Sciences, Hangzhou, China; ^7^ Institute of Metagenomics, Qingdao-Europe Advance Institute for Life Sciences, BGI-Qingdao, Qingdao, China

**Keywords:** immune checkpoint therapy, anti-PD-1, lung cancer, gut microbiome, biomarker

## Abstract

**Background:**

Programmed death 1 (PD-1) and the ligand of PD-1 (PD-L1) are central targets for immune-checkpoint therapy (ICT) blocking immune evasion-related pathways elicited by tumor cells. A number of PD-1 inhibitors have been developed, but the efficacy of these inhibitors varies considerably and is typically below 50%. The efficacy of ICT has been shown to be dependent on the gut microbiota, and experiments using mouse models have even demonstrated that modulation of the gut microbiota may improve efficacy of ICT.

**Methods:**

We followed a Han Chinese cohort of 85 advanced non-small cell lung cancer (NSCLC) patients, who received anti-PD-1 antibodies. Tumor biopsies were collected before treatment initiation for whole exon sequencing and variant detection. Fecal samples collected biweekly during the period of anti-PD-1 antibody administration were used for metagenomic sequencing. We established gut microbiome abundance profiles for identification of significant associations between specific microbial taxa, potential functionality, and treatment responses. A prediction model based on random forest was trained using selected markers discriminating between the different response groups.

**Results:**

NSCLC patients treated with antibiotics exhibited the shortest survival time. Low level of tumor-mutation burden and high expression level of HLA-E significantly reduced progression-free survival. We identified metagenomic species and functional pathways that differed in abundance in relation to responses to ICT. Data on differential enrichment of taxa and predicted microbial functions in NSCLC patients responding or non-responding to ICT allowed the establishment of random forest algorithm-adopted models robustly predicting the probability of whether or not a given patient would benefit from ICT.

**Conclusions:**

Overall, our results identified links between gut microbial composition and immunotherapy efficacy in Chinese NSCLC patients indicating the potential for such analyses to predict outcome prior to ICT.

## Introduction

Immune checkpoint therapy (ICT) represents an option for blocking immune evasion-related pathways elicited by tumor cells (1). Cytotoxic T lymphocyte-associated antigen 4 (CTLA-4) (2), programmed death 1 (PD-1), and the ligand of PD-1 (PD-L1) (3) are the three most studied targets for ICT. Even though ICT has proven successful in the treatment of several types of cancers, the percentage of positive responses varies considerably and is typically in the range of 10% to 47% in different groups of patients (3–5). Previous studies have shown that high tumor mutational burden (TMB) as well as the human leukocyte antigen (HLA) type are correlated with ICT response rate (4, 5). Moreover, convincing evidence has been presented showing that the composition and functional properties of the gut microbiota may influence the efficacy of ICT in Caucasian patients, suggesting that analysis of the gut microbiota in combination with other biomarkers might allow for identification of responders versus non-responders prior to initiation of ICT (6–8), thereby enabling a more personalized treatment of patients. However, the individual contributions of such biomarkers have not yet been compared in a single study setting.

In this study, we used shotgun metagenomics sequencing to analyze the baseline composition and changes in the gut microbiota in longitudinally collected fecal samples from advanced non-small cell lung cancer (NSCLC) patients during anti-PD-1 therapy to initially define the most robust gut microbial-based response predictors. We then compared the identified microbial-based biomarkers with models involving other patient-specific markers such as TMB and blood lymphocyte counts. Based on the dynamic metagenomic profiling of the gut microbiota during anti-PD-1 treatment, we identified several bacterial species and selected functions to be valuable predictors of the response to anti-PD-1 therapy in Chinese NSCLC patients. Finally, we examined to what extent our findings in the Chinese patients could be replicated in a French cohort. Overall, our study suggests that gut microbiome biomarkers may serve as independent predictors of ICT responsiveness also in Chinese NSCLC patients.

## Results

### Diverse Host Factors Influence the Responsiveness to Anti-PD-1 Therapy

After 3 months of treatment of the 85 eligible patients, 13 (15%) were assessed as patients with partial response (PR), 24 (28%) as patients with stable disease (SD), 43 (51%) as patients with progressive disease (PD), and 5 patients (6%) as patients who succumbed to fast death (FD). Of all patients, 31 (36%) had progression-free survival (PFS) beyond 3 months and were regarded as ICT responders (Rs), and the remaining 54 patients (64%) were characterized as non-responders (NRs). The clinical characteristics of the patients are provided in **Table S1**.

**Figure 1:**
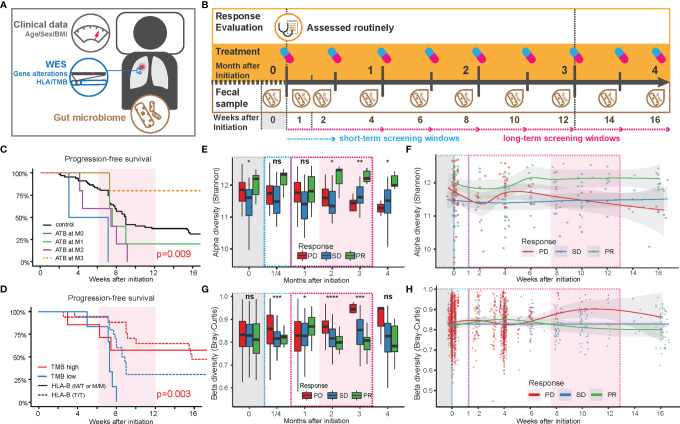
Factors influencing the anti-PD-1 responsiveness in Chinese NSCLC patients. **(A)** Sample collection pipeline, including anthropometrics (age, sex, and BMI), tumor mutations based on somatic tumor and normal tissue, blood, and fecal samples. **(B)** Collection timeline. After 3 months of routinely/regularly administration and response evaluation (see *Methods*), patients were grouped based on partial response (PR), stable disease (SD), or progressive disease (PD), according to the criteria in RECIST 1.1. Fecal samples were collected at the end of each treatment period. Feces from the first week after initiation of treatment (W1) was collected within 3 days after the first treatment. **(C)** Comparison of progression-free survival of patients with or without antibiotics before and after treatment. **(D)** Comparison of progression-free survival of patients with TMB above or below 5.6 (red or blue lines) in patients with a high level of HLA-E type (HLA-B rs1050458 Met/Thr or Met/Met, solid lines) or low level (HLA-B rs1050458 Thr/Thr, dashed lines) of HLA-E type. **(E)** Changes in alpha diversity in the gut microbiota over time in each response group, displayed as a box plot where MIN and MAX corresponds to 9.59 and 12.7, respectively. Significance level (Kruskal–Wallis): **** for p < 0.0001, *** for p < 0.001, ** for p < 0.01, * for p < 0.05 and ns for non-significant. **(F)** Changes in alpha diversity in the gut microbiota over time in each response group, displayed with smooth-fit lines. The p-values of the longitudinal group comparison (splinectomeR) were 0.503 for PD vs. SD, 0.031 for PD vs. PR, and 0.005 for SD vs.PR. **(G)** Changes in beta diversity (Bray–Curtis dissimilarity) in the gut microbiota over time in each response group, displayed as a box plot where MIN and MAX correspond to 0.54 and 0.99, respectively. Significance level (Kruskal–Wallis): **** for p < 0.0001, *** for p < 0.001, ** for p < 0.01, * for p < 0.05 and ns for non-significant. **(H)** Changes in beta diversity in the gut microbiota over time in each response group, displayed with smooth-fit lines. The p-values of the longitudinal group comparison (splinectomeR) were 0.114 for PD vs. SD, 0.239 for PD vs. PR and 0.042 for SD vs.PR.

Similar to the findings by Routy et al. (6), the PFS among patients treated with antibiotics (ATB) within 2 months after the first treatment (n = 12) was significantly lower than for those not treated with ATB (**Figure 1C**). Patients with the highest TMB values (TMB >5.6, n = 24) demonstrated longer PFS than those with lower TMB (**Figure 1D**).

We further found a strong interaction between TMB and HLA-E types. HLA-E has been reported to play a specialized role in cell recognition by natural killer cells (NK cells). When CD94/NKG2A or CD94/NKG2B is engaged, it induces an inhibitory effect on the cytotoxic activity of the NK cell to prevent lysis of target cells. Thus, HLA-E on the cell surface might block immunotherapy through inhibition of NK cell activity (9). In our data, patients with a high expression level of HLA-E (HLA-B rs1050458 Met/Thr or Met/Met) combined with low TMB (n = 6) exhibited the shortest PFS (**Figure 1D**). However, when overall survival was recorded during follow-up, no significant differences were observed in relation to ATB treatment, TMB, and HLA-E type, suggesting that these factors only influence PFS during a relatively short period of time in these advanced NSCLC patients (**Figure S1**).

We next examined the influence of the gut microbiota composition on the categorical response types (PD, SD, PR). For all microbiota-based analyses, we excluded the 12 patients receiving ATB treatment during ICT (**Table S1**). Among the eligible 73 patients, we collected fecal samples longitudinally at biweekly intervals, resulting in a total of 285 samples that underwent shotgun metagenomics sequencing (**Figure S2**). We first mapped the high-quality reads to the integrated gene catalog [IGC (10)] to examine gene diversity differences among the three patient categories (**Figures 1E**–**H**). The alpha diversity showed distinct temporal changes within each category (**Figures 1E, F**). During the first 2 weeks, the diversity in the gut microbiota of the PD and PR groups decreased, then increased during the following 2 weeks. After this period, the alpha diversity in the PD group decreased continuously, while the alpha diversity of the PR group increased to relatively higher values (**Figure 1F**). The SD group maintained a relatively stable alpha diversity during the period of ICT. The diversity differences between the three groups became significant after 2 months of ICT (**Figure 1E**). The beta diversity (Bray–Curtis dissimilarity) also revealed consistent temporal trends. Within the first month, the beta diversity in the PD group tended to increase during the first 2 weeks, then decreased and finally increased during the following 2 months. Compared with the PD group, the beta diversity of the PR group increased slightly in the first month, then decreased during the next 2 months. The beta diversity of the SD group remained stable during the ICT treatment (**Figures 1G, H**).

Combined, the two diversity measures revealed unique trends in the three response groups: the PD and PR groups exhibited a rapid reaction contributing to a fluctuation in the microbial diversity within the first month after treatment. After that, the gut microbiota structure in the PR group remained more similar within the group, and the diversity persisted to be higher than in the other two groups, while the gut microbiota of the PD group individuals became more different and distinct, and less diverse. The gut microbiota of the SD group remained the same throughout the treatment. Except for BMI, we did not observe other collected clinical characteristics to affect the beta diversity measure (**Table S4**).

We next examined if the gut microbiota differed between the NSCLC patients and age- and sex-matched non-NSCLC controls (11) (**Figure S3A**). For this, we used shotgun metagenomics sequencing and the IGC to identify metagenomic species (12) and then used the DMM model (13) to cluster the samples into gut enterotypes at the bacterial genus level. This resulted in identification of three dominating enterotypes in which type 1 (E1) was mainly driven by *Bacteroides*, type 2 (E2) by a combination of *Faecalibacterium*, *Eubacterium*, and *Clostridium*, and type 3 (E3) by *Prevotella* (**Figure S3B**). We identified a similar distribution into enterotypes between patients and non-NSCLC controls with nearly half of them belonging to E1, and compared with the non-NSCLC cohort, the principal contributors to each enterotype were more similar in NSCLC patients (**Figure S3C**). Of note, we did not observe any association between any of the enterotypes and treatment responses.

### A Subset of Gut Bacterial Species Are Enriched in Patients With a Partial Response to Anti-PD-1 Therapy

**Figure 2:**
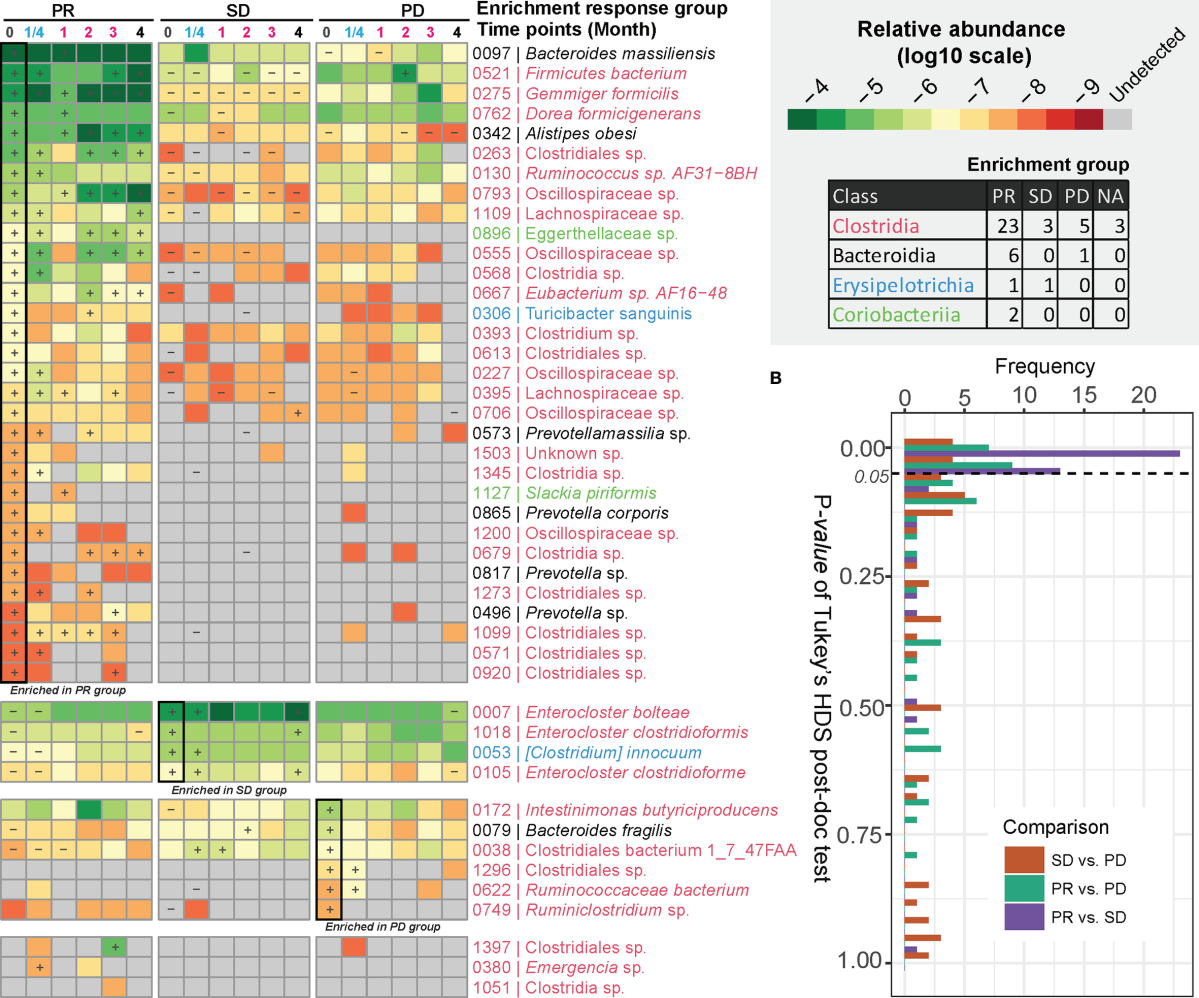
MGSs differentiating anti-PD-1 response groups. **(A)** MGSs that differed significantly in abundance between the three response groups as assessed by ANOVA. The heatmap is colored based on the median value (log10) of relative abundances in each response group. “+” defines the group with the highest abundance of a MGS, and “-” defines the group with the lowest abundance of the given MGS. The bottom three MGSs are marked as “NA” with too low occurrence to define an enrichment group. **(B)** Pairwise comparison of relative abundances of MGSs exhibiting differential enrichment in the response groups. The p value was computed by Tukey’s HSD test.

We next determined to what extent certain bacterial species correlated with the response to anti-PD-1 antibody therapy. After examining BMI-adjusted ANOVA tests for each metagenomic species (MGSs) (12), 45 out of 1,507 MGSs differed significantly in abundance among the three response groups (**Figure 2A** and **Table S5**). Most of these MGSs were annotated to Clostridia (n = 34). The second class was *Bacteroidia* (n = 7), followed by *Erysipelotrichia* (n = 3) and *Coriobacteriia* (n = 2). *Clostridia* was also the predominant class enriched in the PR group (23 out of 32 MGSs). Many of the MGSs were unclassified species that were barely detectable in the SD and the PD group. An individual longitudinal visualization of MGS relative abundance also revealed that these MGSs were not consistently found at all time points (**Figure S4**). In addition, *Bacteroidia* was also enriched in the PR group including the highly abundant species, *Bacteroides massiliensis* (igc0097) and *Alistipes obesi* (igc0342), generally observed in all response groups and present at each time point. *Alistipes obesi*, a newly found member of the *Alistipes* genus, is a gram-negative, motile bacterium with resistance to various drugs (14) which exhibited distinct differences in abundance between the three response groups—with high enrichment in the PR group at baseline, maintaining high levels during treatment, but relatively lower abundance in the SD and PD groups (**Figures 2A** and **S4**). The 4 members of *Prevotellaceae* (igc0573, igc0865, igc0817, and igc0496) were relatively low in abundance and barely observed in the SD and PD groups. Only one member of *Bacteroidia*, *Bacteroides fragilis* (igc0079), was abundant in the PD group at baseline.

Three out of four MGSs enriched in the SD group were assigned to the *Enterocloster* genus, with two of them belonging to *Enterocloster clostridioformis* and one to *Enterocloster bolteae*. Both species are gram-positive, anaerobic opportunistic pathogens associated with various drug-susceptibility patterns (15, 16). All species enriched in the SD group were highly abundant and individually stably represented at each time point (**Figures 2A** and **S4**).

The Tukey’s HDS test showed that most of these MGSs distinguishing the three response groups differed in abundance between the PR and SD groups (**Figure 2B**). This finding is consistent with the diversity analysis and indicates that the PR and SD should not be treated as a single response group, since potential valuable signals might disappear.

### Distinct Gut Bacterial Functionalities Are Enriched in Partial Responders

**Figure 3:**
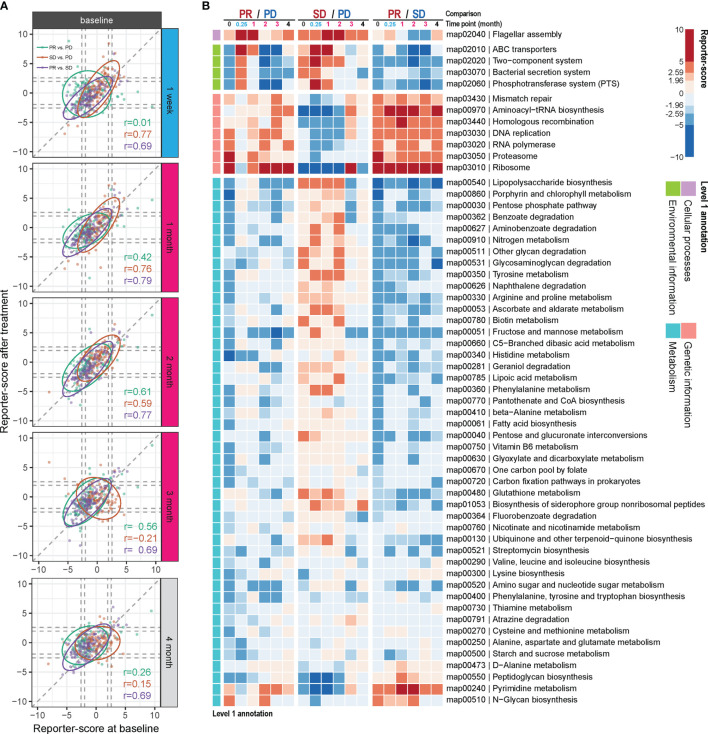
Differential enrichment of gut microbial pathways in the anti-PD-1 response groups. Reporter scores of KEGG-derived pathways based on metagenomics data were computed and compared pairwise between the response groups. **(A)** The functional reporter score obtained from samples collected at different time points after treatment and compared with that of samples collected before treatment (M0). The Spearman correlation indicates the consistency in enriched pathways between samples collected before and after treatment. **(B)** Heatmap of reporter scores for the indicated enriched pathways, grouped based on the designated level 1 pathway level categories. For each combination, a positive value (red) means that this pathway was enriched in the response group with improved response (that is, PR excels over SD and PD; and SD over PD). A negative value (blue) indicates that the pathway was enriched in the other group. The threshold of significance was set at 1.96 (equal to a p value = 0.05) and 2.59 (equal to a p value = 0.01).

We examined if any gut microbial functions were differentially enriched in the PR group as compared to the two other groups. For this, we used a reporter-score pipeline (17) to identify significant differences in the enrichment or depletion of microbiota-related pathways based on KO profiles. The scores obtained at each time point after treatment were further compared to those obtained at baseline. In general, the similarity in scores between the response groups, compared two by two, was relatively stable throughout the ICT treatment from time points M0 to M4, with the biggest dissimilarity observed comparing PR and PD at time point M0 vs. W1 (**Figure 3A** and **Table S6**). This indicates that the contribution from specific functional pathways within the gut microbiota may shift rapidly in the PR group as compared to PD.

Thus, compared to the PD group, one pathway belonging to cellular processes was enriched in the PR group at W1 and M1 and in the SD group at M1 and M2 (map02040; **Figure 3B**). This pathway is related to flagella assembly and bacterial chemotaxis, showing an increased score already within the first week of ICT initiation in the PR group (**Figures S5**, **S6**).

Another pathway that rapidly shifted within the first week of ICT initiation in both the PR and SD groups relative to the PD group is related to membrane transport involved in environmental information processing. One of them encompasses the bacterial secretion system (map03070, **Figures S5**–**S7**), which is related to bacterial interaction with the host immune system, and another is related to ABC transporters (map02010, **Figures S5**, **S6**). However, these changes vanished within the first month in the PR group and after 2 to 3 months in the SD group with a reciprocal increase in the PD group after 2 months of ICT (**Figure 3B**).

Genetic information-related pathways (map03430, 00970, 03440, 03030, 03020, 03050, and 03010) were consistently enriched over time in the PR group, but deficient in the SD group (**Figure 3B**), and the SD group showed to be more divergent from the PR group than the PD group for these functions.

Comparing the PR versus the PD group, we observed that a large fraction of genes involved in the lipopolysaccharide (LPS) pathway (map00540) was enriched in the PD group at baseline, and at week 1, week 2, and month 4 following initiation of ICT (**Figure S8A**). However, we also observed that at the same time points LpxM tended to be relatively enriched in the PR group. LpxM encodes the enzyme adding the sixth acyl chain to the lipid A molecule of LPS and is reported as the essential enzyme for production of the hexa-acylated LPS molecule that can activate human TLR4 (18), while the expression of the other enzymes in the LPS pathway could produce a penta-acylated LPS molecule, which is a poor activator of human TLR4 (19, 20), suggesting different potential for activation of TLR4 in the PR and PD groups.

The remaining bacterial functions that exhibited significantly different enrichment between the response groups were related to metabolism. The reporter scores from this group were relatively modest and were generally reduced in the PR group (**Figure 3B**), except for pyrimidine metabolism (map00240) and N-glycan biosynthesis (map00510), which remained consistently enriched in the PR group.

Combined, the metagenomics and functional enrichment results indicated that a number of gut bacteria changed during the course of ICT and that this might underlie the identified functional response differences between the three treatment response groups.

### Gut Microbiota Taxonomy and Function as Predictors of ICT Efficacy

**Figure 4:**
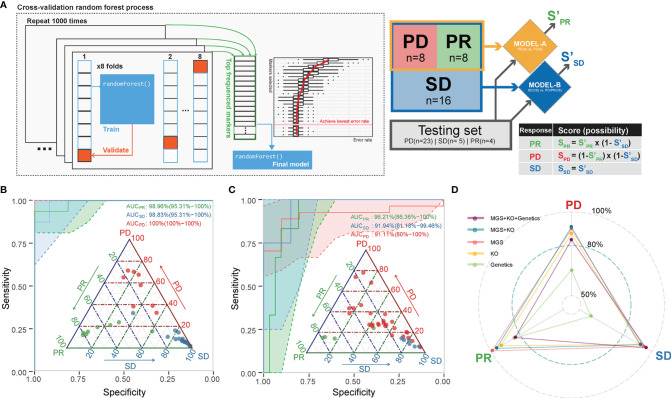
A combined random forest classifier for predicting anti-PD-1 response outcome based on gut microbiota, tumor, and immune data. **(A)** A two-tiered random forest process with cross-validation for model training was implemented. The method includes two random forest models: The first was trained to distinguish PR and PD. The second was designed to distinguish SD from all the rest. The predicted probability score was weighted by the two models as defined. **(B)** The classification of the response groups in the training set. All three response groups achieved an AUC above 98.8%. Confidence intervals (CIs) of 95% are shown in parentheses. Shadowed areas were computed by a SE of 95% CI. **(C)** The classification of the response groups in the test set. All three response groups achieved an AUC above 91%. Confidence interval (CIs) of 95% are shown in parentheses. Shadowed areas were computed by a SE of 95% CI. **(D)** Adding tumor mutation information (including TMB, EGFR, and ALK mutation type) into the prediction model did not improve the performance. The AUC of the predicting ability for the three response groups in the testing set is shown in the radar plot.

We next aimed to use the identified microbiome-based MGS and KO markers at baseline as candidate biomarkers to build a model predicting the likelihood that patients would benefit from ICT prior to administration. Since PRs, SDs, and PDs might be related to different microbial markers, we selected a strategy based on a two-tiered random forest model, where first PD and PR were separated in model A followed by a separation of SD from PD/PR in model B (**Figure 4A**; details described in *Methods*). The training set was able to distinguish samples from each of the response groups very well (AUC = 1 for all, **Figure 4B** and **Table S7**). To avoid overfitting and to validate the performance, all the remaining samples were included as a testing set. For each test sample, we calculated the predicted probability being correctly assigned into PD, SD, or PR. This resulted in an area under the curve (AUC) larger than 0.9 for each of the three response groups (**Figure 4C**), implying that the baseline gut microbiota, when including both taxonomy and function, provides a fairly good predictor of whether or not an advanced NSCLC patient would benefit from ICT.

### Performance of Prediction Models Including Genetic Markers

We further attempted to improve the prediction performance by including additional host data comprising genetics data including TMB, EGFR, and ALK mutations (**Table S2**) of relevance for ICT. We found that the combined microbiome MGS profile and whole exon sequencing (WES) data model resulted in a generally better performance, as SD and PR predictions achieved an AUC of 0.94 for SD and an AUC of 0.96 for PR by inclusion of WES data. On the other hand, we found that the microbiome-based model (MGS and KO) performed best in predicting the PD outcome, as the AUC decreased from 0.81 to 0.77 by inclusion of the other data sets (**Figure 4D**).

### Comparison Between Metagenomic Profiles in Chinese and French NSCLC Cohorts

To estimate the generalization of our findings, we compared the previously published French cohort (6) to the Chinese cohort. For this comparison, we selected the NSCLC cases from the French cohort, as we found the metagenomics profile in patients with different tumor types to be significantly different based on a PERMANOVA test (data not shown). Quality controls and statistical tests were conducted on the French cohort using the same bioinformatics pipelines as were used for the Chinese cohort. Outcomes independently calculated for each cohort were then compared.

**Figure 5:**
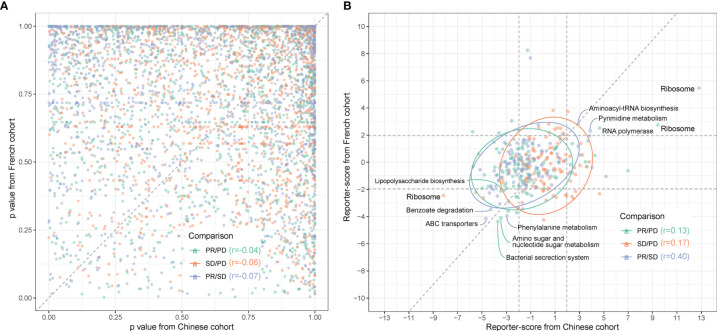
Comparison between gut microbiota predictors in Chinese and Caucasian NSCLC cohorts. Metagenomic sequencing data from a publicly accessible French NSCLC cohort (6) were processed for generation of MGSs and functional pathway analysis (n = 40, individuals using ATB were excluded). **(A)** QQ plot of the similarity between MGSs in the three response groups (pairwise comparisons) in Chinese (x-axis) and French (y-axis) patients. p-values were calculated by ANOVA. **(B)** The reporter scores obtained from functional pathway analysis as in **Figure 3** were compared pairwise in the response groups in the Chinese (x-axis) and French (y-axis) patients. Significant positions (> ± 1.96) were defined by the dashed squares. Pathways that are significantly enriched in one response group are named and colored according to the enriched response group as indicated in the figure.

When comparing the MGS profiles in the three response groups PD, SD, and PR using ANOVA with *post-hoc* Tukey’s HSD test, we found a surprisingly low correlation between the *p-*values for each pair-wise group comparison in the two cohorts (**Figure 5A**).

Functional-level comparisons showed a higher correlation, and we found several of the previously mentioned microbial pathways in the Chinese cohort to also characterize the French cohort, such as the bacterial secretion system (map03070), ABC transporters (map02010), and LPS biosynthesis (map00540) (**Figure 5B**). We also identified the same enrichment for bacteria containing LpxM in the French PR group (**Figure S9**).

We next examined if the discrepancies between the two cohorts at the bacterial species level might rely on the presence of different strains and, thus, identification of different MGSs in the two cohorts. Since *A. muciniphila* was previously reported to be enriched in the response group of the French cohort (6), we addressed this question specifically for *A. muciniphila*. When grouping the cohorts into Rs and NRs based on PFS at the third month (PFS 3mo) and comparing the occurrence of MGSs assigned to *A. muciniphila*, we found that the occurrence of each MGS was similar in the two cohorts. However, *A. muciniphila* seemed more enriched in Rs in the French cohort, especially for MGS.igc0776 (**Figure S10A**). Further statistical analysis comparing Rs and NRs showed that the significantly enriched *A. muciniphila* MGSs in the two cohorts were different. The *A. muciniphila* significantly enriched in Rs of the French cohort belongs to MGS.igc0118, while in the Chinese cohort MGS.igc0776 was enriched in Rs (**Figure S10B**). This indicates that the differences between cohorts in relation to the response-critical *A. muciniphila* might be due to colonization by different strains.

## Discussion

In this study, we performed a comprehensive characterization of temporal metagenomic-based and clinical biomarkers in Chinese patients with advanced NSCLC and their response to ICT. We noticed that the relative abundance of specific species exhibited robust trends in the different response groups indicating the resilience of these species during treatment, also reflected in robust trends of selected functional pathways (**Figures S4**, **S11**). In keeping with previous studies, we found that TMB as well as specific HLA types influenced the response to ICT in this Chinese cohort. It is now recognized that the gut microbiota impacts on the effect of drug treatment by modulating both drug metabolism and toxicity (21, 22), and recent pioneering work has revealed possible causal relationships between the gut microbiota and the outcome of ICT for NSCLC, metastatic melanoma, and renal cell carcinoma (7, 23, 24) However, in these studies enrichment of different bacteria was identified to characterize Rs versus NRs. It has been speculated to which extent differences in relation to marker species in these studies were related to confounding factors such as sampling, DNA extraction, DNA sequencing, different analytical pipelines, or if these differences reflected distinct cohorts or cancer types (25).

The recently published reanalysis of so far published data concluded that the differences were not due to different analysis pipelines but failed to recapitulate a number of the findings concerning marker species in the different studies (25). However, an enrichment of *A. muciniphila* seemed to characterize Rs in the three analyzed studies and another study involving a Chinese HCC cohort (26). Another Chinese NSCLC study also reported an enrichment of *A. muciniphila*, but since this study employed 16S rRNA amplicon sequencing, it is not clear which species or strains might be involved (27).

Therefore, we examined if the discrepancies between the Chinese and French cohorts at the bacterial species level might rely on the presence of different strains, and thus, identification of different MGSs in the two cohorts. Since *A. muciniphila* was previously reported to be enriched in the response group of the French cohort (6), we addressed this question specifically for *A. muciniphila*. We found that the occurrence of each MGS was similar in the two cohorts. However, *A. muciniphila* seemed more enriched in Rs in the French cohort, especially for MGS.igc0776 (**Figure S11A**). It is noteworthy that we observed an enrichment of *A. muciniphila* in Rs in the Chinese cohort, despite the otherwise pronounced differences observed in the composition of the gut microbiota in Caucasian and Chinese individuals (10). This finding mirrors the finding that despite considerable differences in the composition of the gut microbiota in European and Chinese individuals, enrichment of a number of bacterial species characterized individuals with colorectal cancers in both ethnic groups (28).

Comparisons at the functional level also demonstrated that changes in the relative abundance of genes involved in several microbial pathways in the Chinese cohort were recapitulated in the French cohort, including bacterial secretion system (map03070), ABC transporters (map02010), and LPS biosynthesis (map00540), where we also observed an enrichment of bacteria harboring *LpxM* in the PR groups in both the Chinese and French cohorts. A finding suggesting that capacity for production of the hexa-acylated form of LPS may improve ICT outcome, perhaps *via* activation of TLR4, the innate immune receptor for LPS.

Importantly, in spite of the limited sample size, and the fact that three different anti-PD-1 antibodies were used for treatment of the NSCLC patients, we were able to build models based on MGS and KO markers at baseline predicting the probability that a given patient would benefit from ICT with an AUC larger than 0.8 for each of the three response groups, hence pointing to the value of including both microbial taxonomy and function at baseline for prediction of the ICT response outcome in advanced NSCLC patients prior to initiation of the treatment. However, to generalize this model, more samples are required for both training and validation. In addition, the possible impact of the use of different anti-PD-1 antibodies should be investigated in future studies, and more information on prior treatment and possible dietary preferences and intake would be desirable. The lack of lifestyle information in the real-life clinical setting of the present study is a limitation, but still, we envisage that the strategy of the computational-based training process described here will be of value for the generation of more robust prediction models in the future.

In conclusion, our results delineate specific links between gut microbial composition and immunotherapy efficacy in Chinese NSCLC patients. The consistency between compositional and functional properties of the gut microbiota between the French and Chinese cohorts in predicting the outcome of ICT supports the notion that such analyses may be developed into a powerful tool predicting outcome prior to initiation of ICT, and the published mouse studies even suggest that supplementation with specific bacteria may improve treatment (6–8). Evidently, much larger cohorts of different ethnicity are needed, but results so far are promising.

## Methods

### Patient Characteristics and Clinical Trial Design

NSCLC patients were treated with anti-PD-1 monotherapy agents at Sun Yat-sen University Cancer Center between December 2015 (the first date on which a patient with NSCLC was treated) and August 2017 (the last date to initiate therapy). All patients were treated as part of a clinical trial program (registered with ClinicalTrials.gov, NCT02593786 and NCT02613507 for the nivolumab monotherapy trial, NCT02721589 for the camrelizumab monotherapy trial, and NCT02835690 for the pembrolizumab combination trial; more details in **Table S1**). Eligible patients for this study were selected based on the following criteria: (i) >18 years old; (ii) Eastern Cooperative Oncology Group performance status: 0 or 1; (iii) with histologically or cytologically confirmed NSCLC that were clinically advanced or recurrent; (iv) failure after first-line platinum-based doublets chemotherapy; (v) measurable disease per Response Evaluation Criteria in Solid Tumors version 1.1; and (vi) life expectancy of ≥3 months. Patients with the following criteria were excluded: (i) with prior malignancy (except for non-melanoma or certain *in situ* cancers, or complete remission ≥2 years); (ii) patients had active or a history of autoimmune disease; (iii) were in medical conditions requiring the use of immunosuppressive medications including steroids; and (iv) active central nervous system metastases (except previously treated, stable brain metastases without progression ≤4 weeks or steroid therapy ≤14 days before initiating study treatment). Computed tomography (CT) or magnetic resonance imaging (MRI) scans were reviewed by the clinical investigators. Progression-free survival (PFS) was defined as the time from the beginning of treatment to the date of progressive disease (PD). Patients who did not progress were evaluated at the date of their last scan. The objective response rate was defined as the percentage of patients with complete response (CR) or partial response (PR).

Patients terminated any other therapies 2 weeks before receiving PD-1 mAb (pembrolizumab, camrelizumab, also known as SHR-1210 or nivolumab, also known as Opdivo). Patients received standard doses of nivolumab (240 mg) or camrelizumab (200 mg) once every second week, or pembrolizumab (200 mg) once every third week. In this study, 85 patients were eligible recruited; 12 were excluded in microbiota-based analyses as they received antibiotic treatments during administration.

Responses to treatment were assessed by the site investigator using RECIST 1.1 with CT or MRI scans obtained at baseline and every 8 weeks for Nivolumab and Camrelizumab, every 6 weeks for Pembrolizumab during treatment. A landscape of this study design is shown in **Figures 1A, B**. The overall treatment schemes and response types are provided in **Table S1**.

### Blood Collection and Lymphocyte Phenotype Analyses

Fasting blood was collected at baseline and every time before receiving PD-1 mAb. Heparin plasma samples were collected for lymphocyte analysis. Anti-CD45-PerCP-Cy™5.5 was used to gate lymphocytes, anti-CD3-FITC for the identification of T lymphocytes, anti-CD4-PE-Cy™7 for detecting T-helper/inducer lymphocytes, CD8-APC-Cy7 for the identification of suppressor/cytotoxic T lymphocytes, CD19-APC to identify B lymphocytes, and CD16-PE and CD56-PE for the identification of natural killer (NK) lymphocytes by using the BD Multitest™ 6-color TBNK reagent (Catalog No 644611; BD Biosciences, San Jose, CA, USA). The cells were analyzed by a FACSCalibur flow cytometer using BD FACSDiva clinical software (BD Biosciences) as indicated by the manufacturer. The results corresponding to each lymphocyte type are presented as percentages in human peripheral blood (**Table S2**).

### Whole Exon Sequencing for Tumor Tissue

Tumor biopsies were collected before treatment initiation. Genomic DNA from tumor biopsies was extracted using the DNeasy Tissue Kit (Qiagen, Germantown, MD, USA). Genomic DNA from peripheral blood was extracted using DNeasy blood and tissue kits (Qiagen, USA) as normal control. The extracted genomic DNA was fragmented into ~250 bp by using an M220 Focused-ultrasonicator (Covaris, Brighton, UK). DNA libraries were prepared using the HyperPrep Kit (KAPA Biosystems Inc., Wilmington, MA, USA), followed by exome capture using the Agilent V6 Kit (Agilent Technologies, Inc., Santa Clara, CA, USA). Finally, the whole exome DNA libraries were sequenced using the Illumina HiSeq 4000 platform with paired 150-bp reads. Sequencing data were generated to target the mean coverage of ~200× for the tumor biopsies and ~60× for the normal control.

WES data were processed to variant detection as previously described (29). Briefly, paired-end sequencing data were aligned to the reference human genome (build hg19) using the Burrows-Wheeler Aligner (bwa-mem). MuTect was performed to pair normal and tumor BAM files and identify somatic single-nucleotide variants (SNVs) of tumor with default parameters. Oncotator was applied for somatic SNV annotation. Four exclusion filters were applied for somatic SNV calling: (i) less than 5 alternative reads in tumor samples; (ii) less than 5% variant allele frequency (VAF); (iii) less than 15 reads in total in the tumor and control samples; and (iv) presence of the variant in the 1000 Genomes project at a frequency >1%. Tumor mutational burden (TMB) was defined as the number of somatic, coding, base substitution, and indel mutations per megabase of genome examined, according to the method of Chalmers et al. (30). The TMB profile of each patient is provided in **Table S2**.

### Human Leukocyte Antigen Typing

HLA types were predicted from WES data by the previously described HLA typing method (31). The HLA-I alleles were classified into twelve supertypes based on similar peptide-anchor-binding specificities (32, 33).

### Gut Microbiome Metagenomic Analysis

#### Sample Collection

Fecal samples were collected at the hospital and frozen immediately at -80°C. A total of 285 fecal samples were collected before initiation until the end of ICT. For each patient, the qualified sample collected closest to the next treatment was selected as the included sample. All samples were shipped to the China National GeneBank (CNGB) for sequencing according to the Sample Delivery Suggestions (No. CNGB-DP-SOP16-002/A2).

#### DNA Library Preparation and Sequencing

DNA libraries were prepared by using 500 ng of input DNA. DNA was ultrasonically fragmented by using a E220 Focused-ultrasonicator (Covaris, UK), yielding 300–700-bp fragments. Products were purified with an AxyPrep Mag PCR Clean-Up Kit (Axygen Scientific, Inc., Union City, CA, USA) and eluted with 45 μl TE buffer. Afterward, 20 ng of purified DNA was processed with end-repairing and A-tailing by using a 2:2:1 mixture of T4 DNA polymerase (ENZYMATICS™ P708-1500), T4 polynucleotide kinase (ENZYMATICS™ Y904-1500), and rTaq DNA polymerase (TAKARA™ R500Z). Adaptors with specific barcodes were ligated to the DNA fragment by T4 DNA ligase (ENZYMATICS™ L603-HC-1500) at 23°C, followed by PCR amplification. Finally, a single-strand circular DNA library was generated using 55 ng of purified PCR products by denaturing at 95°C and circular ligation using T4 DNA ligase (ENZYMATICS™ L603-HC-1500) at 37°C. Equal amounts of 8 barcoded libraries were pooled for the generation of DNA Nanoballs (DNB) and loaded onto one lane for sequencing using the BGISEQ-500 platform.

Sequencing was performed according to the BGISEQ-500 protocol (No. CNGB-DP-SOP10-002) employing the SE50 mode with a following base calling process to remove adaptors automatically (34). Finally, 285 samples were successfully sequenced and generated 84.53 ± 23.18 million reads per sample (**Table S3**).

Raw reads were quality controlled, host reads removed, and the bacterial reads aligned to the integrated gene catalogue (IGC) 9.9M reference to obtain a normalized gene abundance profile, as described previously (10). The metagenomic species (MGS) profile was generated based on a previously described procedure (12). Annotation of MGSs was updated with extra information by a random forest-based method and curated manually.

### Statistical Analysis

Statistical analyses were mainly performed in the program R version 3.4.3 with the following packages: Spearman correlation was performed by *cor()*; ANOVA and Tukey’s HSD tests were performed by *avo()* and *TukeyHSD()*, respectively; PERMANOVA was performed based on *adonis()* from the vegan package (35); principal component analysis (PCA) was performed by *prcomp()*; and *Pathview* was used for pathway visualization (36). Alpha diversity was calculated as the Shannon index (37), while Bray–Curtis dissimilarity (38) was used to compute the beta diversity. SplinectomeR was used for the longitudinal microbiome group comparison (39). The number of enterotypes was determined using the DMM model (13). The method used to calculate the reporter score has been described previously (40). All missing data were left as NA and not imputation method used.

### Prediction Model

Differences in TMB, MGS, and/or KO relative abundances between response groups were used as candidate predictors. Two random forest models (41) were combined to predict the response outcome. The first model (model A) was trained to provide a predicted probability of PR and PD. 8 PR and 8 PD patients were randomly selected as the training set. The second model (model B) was used to provide a predicted probability of SD or not. 16 SDs combined with the model A training set (8 PRs and 8 PDs sum as 16 non-SDs) were used for training. For each model, 8-fold cross-validation processes were repeated 1,000 times. For each repetition, a random forest decision tree was built by randomly picked features (MGS and/or KOs) from 7-fold samples. The remaining one-fold was used to test the performance of this decision tree. The performance was determined for each feature used. When the cross-validation process was terminated, the features that contributed the most were selected as candidate biomarkers to generate a determined model for training model A or model B. Finally, the probability for each response group was obtained by multiplying the probability from model A and that from model B. All remaining patients who were not included for training (n(PD) = 23; n(SD) = 5; n(PR) = 4) were used as the testing set for validation. The area under the curve (AUC) of sensitivity over specificity was used to estimate the performance of the prediction outcomes. This method is illustrated in **Figure 4A**. Since TMB and lymphocyte data were not fully aligned with the fecal samples used for metagenomics, the multi-omics-based prediction models were first trained based on these datasets separately and finally combined to determine the multiple test model prediction probabilities.

## Data Availability Statement

Public metagenomic sequencing data from the French advanced NSCLC patient cohort are available from the European Nucleotide Archive (EMBL-EBI) under accession number PRJEB22863. Metagenomic sequencing data of fecal samples for the non-NSCLC Chinese cohort have been deposited into CNGB Sequence Archive (CNSA) (42) of the China National GeneBank DataBase (CNGBdb) (43) with accession number CNP0000175. Metagenomic sequencing data for 285 fecal DNA samples from the Chinese advanced NSCLC patient cohort have been deposited into CNGB Sequence Archive (CNSA) of the China National GeneBank DataBase (CNGBdb) with accession number CNP0000636.

## Ethics Statement

The studies involving human participants were reviewed and approved by the Ethics Committee of Cancer Center of Sun Yat-sen University. The patients/participants provided their written informed consent to participate in this study.

## Author Contributions

LZ, KK, SB, YoH, JL, and CF designed and coordinated the study. FG oversaw the establishment of this patient cohort. CF, FG, HZ, SQ, WF, and SL curated the phenotype data. CF and HZ performed the bioinformatic analyses of metagenomic data. SQ and LX performed the analysis of tumor mutation data. CF performed the bioinformatic analysis of lymphocyte data. HR provided guidance for statistical methods. CF prepared the figures and wrote the first version of the manuscript. JM revised the taxonomic annotations. SB, KK, DW, HZZ, YoH, WF, YaH, and LZ provided a substantial revision of the manuscript. All authors contributed to the article and approved the submitted version.

## Funding

This work was supported by grants from the National Key R&D Program of China (2016YFC0905500, 2016YFC0905503), Chinese National Natural Science Foundation (81772476, 81602005, 81872499, and 81702283), Science and Technology Program of Guangdong (2017B020227001), Science and Technology Program of Guangzhou (201607020031), and Shenzhen Municipal Government of China (No. KQJSCX20180329191008922).

## Conflict of Interest

Authors CF, LX, YoH, HZ, DW, HR, SQ, HzZ, JL, SL, HY, JW, and KK were employed by BGI-Shenzhen.

The remaining authors declare that the research was conducted in the absence of any commercial or financial relationships that could be construed as a potential conflict of interest.

## Publisher’s Note

All claims expressed in this article are solely those of the authors and do not necessarily represent those of their affiliated organizations, or those of the publisher, the editors and the reviewers. Any product that may be evaluated in this article, or claim that may be made by its manufacturer, is not guaranteed or endorsed by the publisher.

## Supplementary Material

Supplementary Figure 1 Overall survival. **(A)**. Overall survival of patients with or without antibiotics before and after treatment. **(B)**, Overall survival comparison in patients with TMB above or below 5.6 (red or blue lines). **(C)**. Overall survival comparison in patients with a high level of HLA-E type (HLA-B rs1050458 Met/Thr or Met/Met, solid lines) or low level (HLA-B rs1050458 Thr/Thr, dashed lines) of HLA-E type.

Supplementary Figure 2 Fecal sample collection scheme. Samples for the first week were collected within 3 days after the first ICT treatment. For each patient, the sample collected closest, but prior, to the next ICT treatment was selected as the representative sample.

Supplementary Figure 3 Comparison of the gut microbiota composition at the enterotypes level between Chinese NSCLC patients and healthy controls. Samples collected at baseline (M0) and the first month (M1) after ICT initiation were selected for enterotype assessment. **(A)**, A healthy age- and sex-matched Chinese cohort was included for background comparison. **(B)**, Enterotype numbers were calculated at the genus level using the DMM model^11^. The three dominating genera for each enterotype are shown, in which type 1 (E1) was mainly driven by *Bacteroides*, type 2 (E2) by *Faecalibacterium*, and type 3 (E3) by *Prevotella*. **(C)**, PCA plot showing the distribution of samples belonging to the three enterotypes.

Supplementary Figure 4 Relative abundance of Individual MGSs at each time point. MGSs that differed in abundance between response groups are visualized. To reveal longitudinal trends, patients donating M3 samples are visualized.

Supplementary Figure 5 Flagellar assembly and related pathways enriched in the PR versus the PD group. Gut microbial pathways where the reporter-score reached statistical significance are listed, displayed as the z-score calculated between the PR and PD groups. A positive value means that the pathway was enriched in the PR group, while negative values indicate an enrichment in the PD group. Pathways were derived from KEGG.

Supplementary Figure 6 Flagellar assembly, bacterial chemotaxis and bacterial secretion system pathway enrichment in partial responders versus disease progressors. Two KEGG pathways, flagellar assembly **(A)** and bacterial chemotaxis **(B)**, both playing a part in cellular motility, contributed with the highest average reporter-score to the difference between PR and PD patients. Most of the enzymes in the flagellar assembly pathway were enriched in the PR group. Bacterial chemotaxis is also related to flagellar assembly and was similarly enriched in the PR group **(C)**. The z-score (color) is based on PR/PD.

Supplementary Figure 7 Time dependent enrichment of KOs belonging to the bacterial secretion system pathway in the PR group. The bacterial secretion system is involved in membrane transport, especially in selected pathogens. Among six types of secretion proteins, type III, type II and type VI were enriched in the PR group during week 1 (W1). Type II is able to secrete intracellular toxins, while type II and III and type VI are both capable of secreting effector proteins into host cells. The z-score (color) is based on PR/PD.

Supplementary Figure 8 Enriched KOs in the LPS pathway in different response groups. **(A)**, Most enzymes in the LPS pathway were enriched in the PD group, resulting in production of penta-acylated LPS, while LpxM was enriched in the PR group, allowing for generation of hexa-acylated LPS by the LpxM-containing bacteria. The pattern in a) existed at baseline, week 1, week 2 and after 4 months of ICT. The z-score (color) is based on PR/PD. **(B)**, Relative abundance (log10 scale) of KOs annotated to LpxM and LpxL at each time point.

Supplementary Figure 9 Enriched KOs in the LPS pathway in the different response groups of a French NSCLC cohort. The reporter-score for each of the LPS pathway enzymes were enriched in a NSCLC French cohort as well as in the Chinese cohort.

Supplementary Figure 10 Occurrence and abundances of *Akkermansia muciniphila* in a French and Chinese NSCLC cohort. *Akkermansia muciniphila* MGSs were identified in the two cohorts and compared. **(A)**, Occurrence of three *A. muciniphila* MGSs across Rs (green) and NRs (red). **(B)**, Abundance of the three *A. muciniphila* MGSs across Rs and NRs in Chinese and French NSCLC patients.

Supplementary Figure 11 Relative abundance of individual pathways at each time point. KOs that differed in relative abundance between the response groups were averaged to reveal longitudinal trends. Only patients donating M3 samples are visualized.

## Abbreviations

PD-1, programmed death 1; PD-L1, the ligand of PD-1; ICT, immune-checkpoint therapy; NSCLC, non-small cell lung cancer; HLA-E, human leukocyte antigen class I histocompatibility antigen, alpha chain E; TMB, tumor mutational burden; PFS, progression-free survival; RECIST, Response Evaluation Criteria in Solid Tumors; BMI, body mass index; DNB, DNA nanoball; KO, KEGG Orthology; TLR4, Toll-like receptor 4; LPS, lipopolysaccharide; RNA, ribosomal ribonucleic acid.
