# Supplementary figures and images for: Distinct Functional Metagenomic Markers Predict the Responsiveness to Anti-PD-1 Therapy in Chinese Non-Small Cell Lung Cancer Patients
Source: Front Oncol. 2022 Apr 21;12:837525. doi: 10.3389/fonc.2022.837525 (PMC9069064; doi:10.3389/fonc.2022.837525)

A

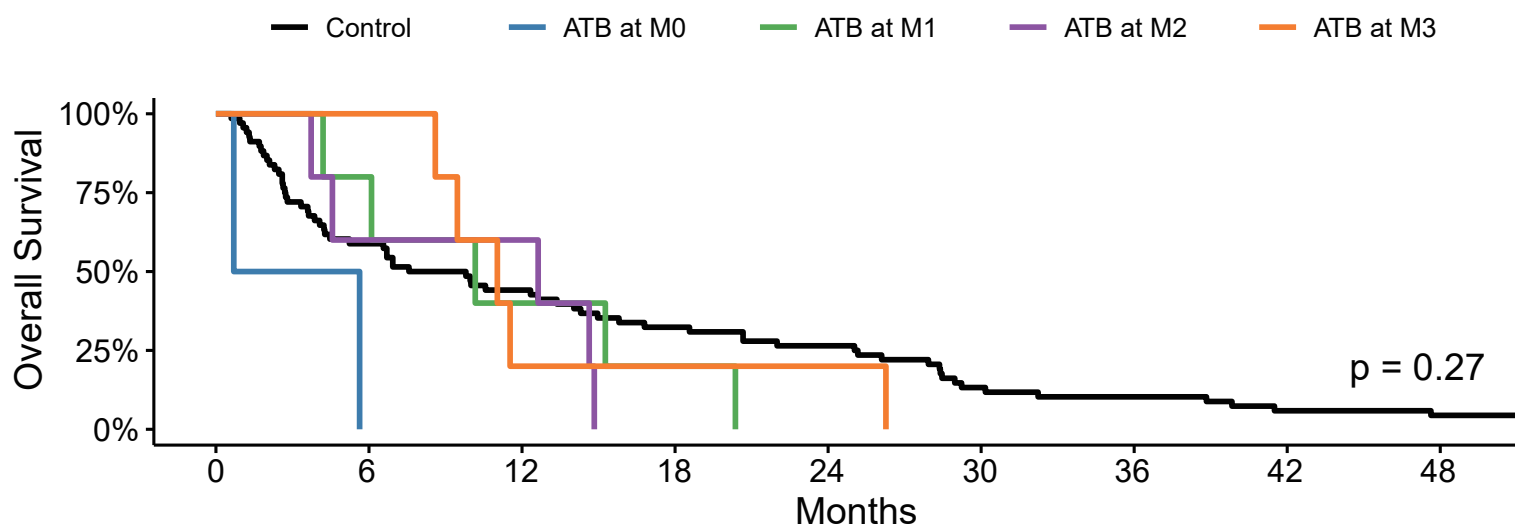

B

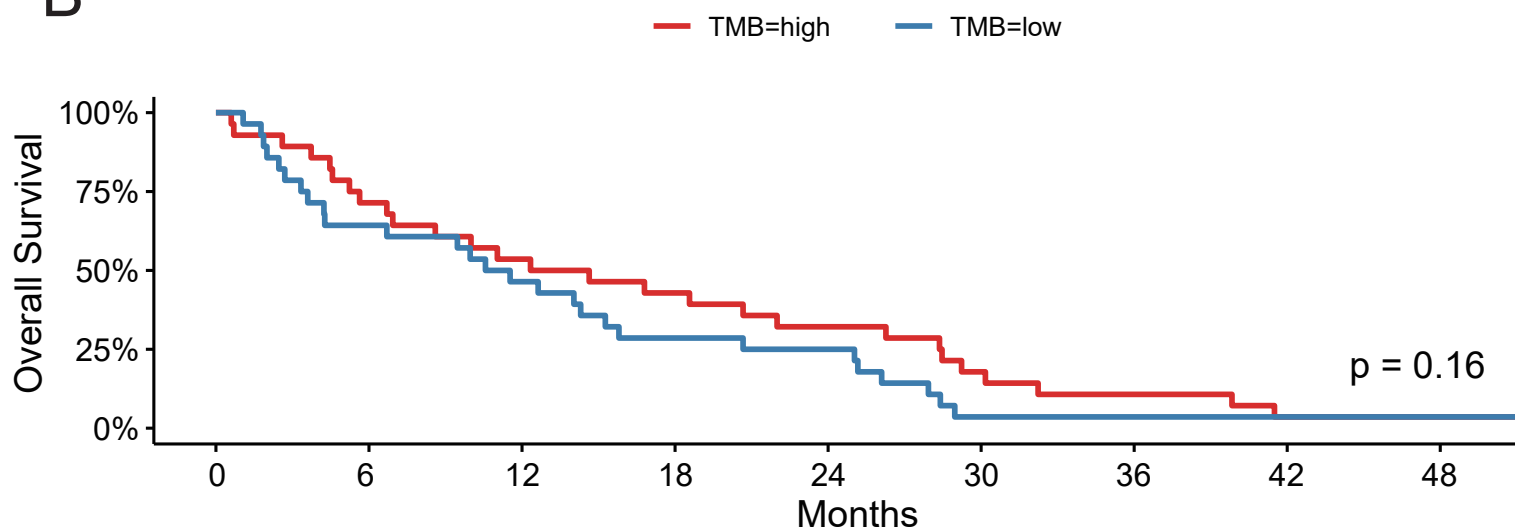

C

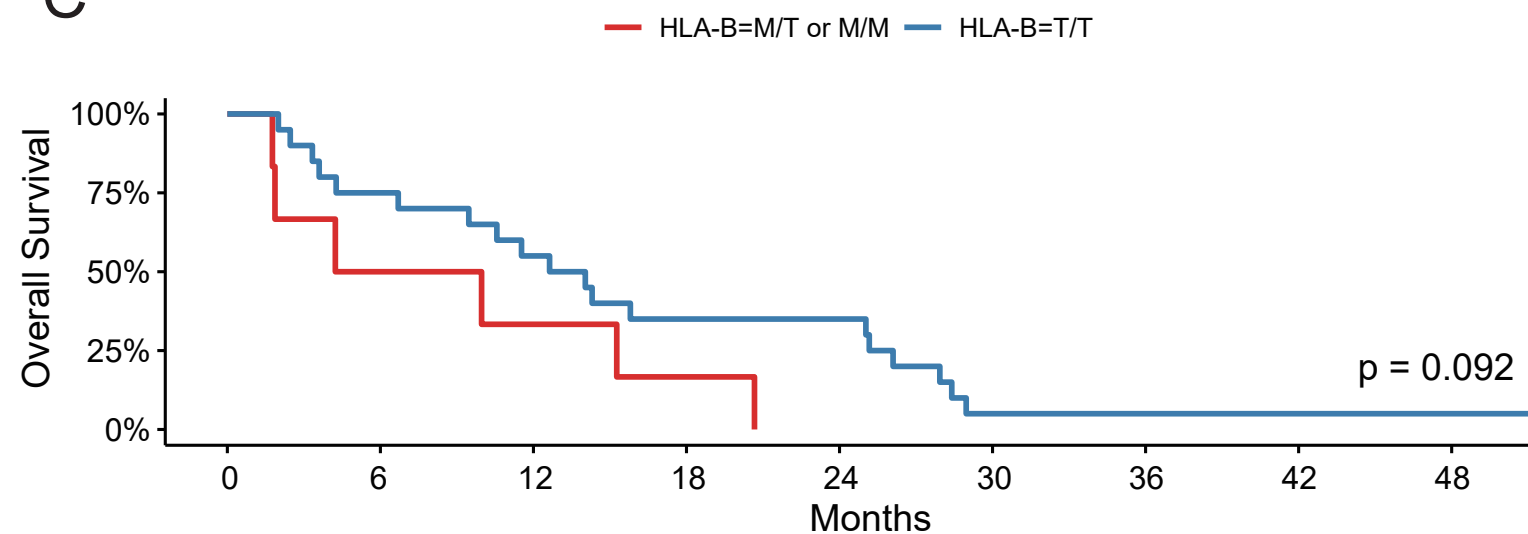

Supplement: Supplementary Figure 1 — Overall survival. (A). Overall survival of patients with or without antibiotics before and after treatment. (B), Overall survival comparison in patients with TMB above or below 5.6 (red or blue lines). (C). Overall survival comparison in patients with a high level of HLA-E type (HLA-B rs1050458 Met/Thr or Met/Met, solid lines) or low level (HLA-B rs1050458 Thr/Thr, dashed lines) of HLA-E type. [file Image_1.pdf]

A

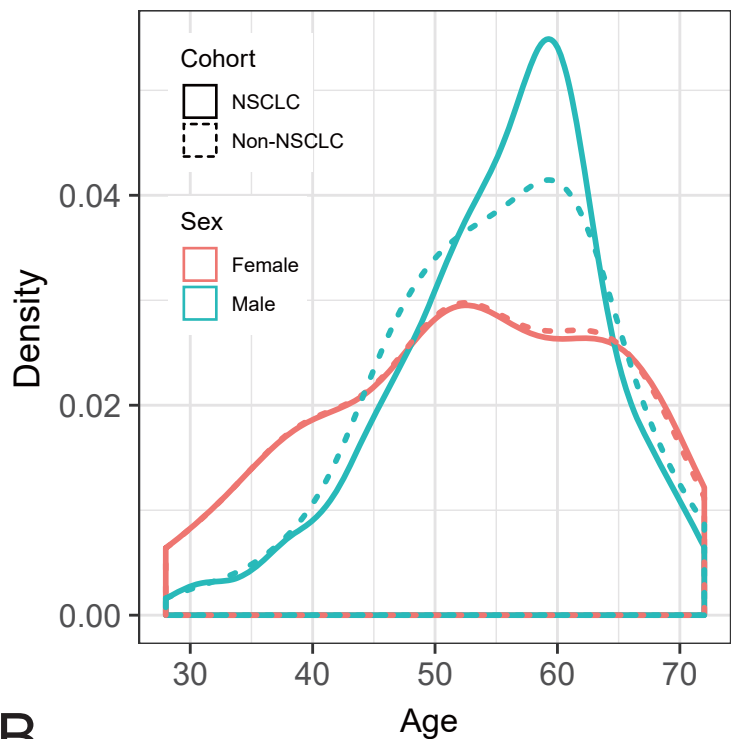

B

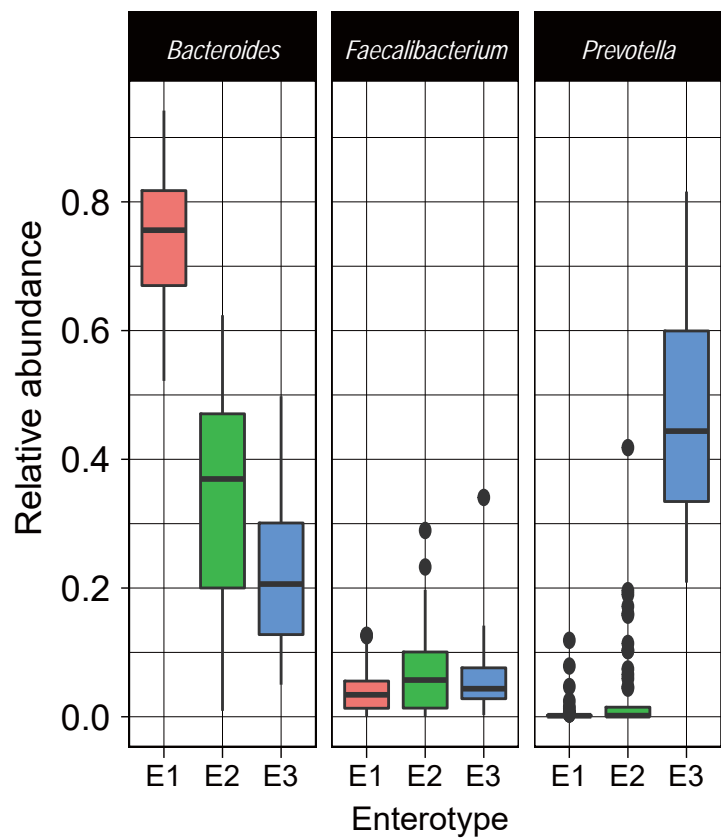

C

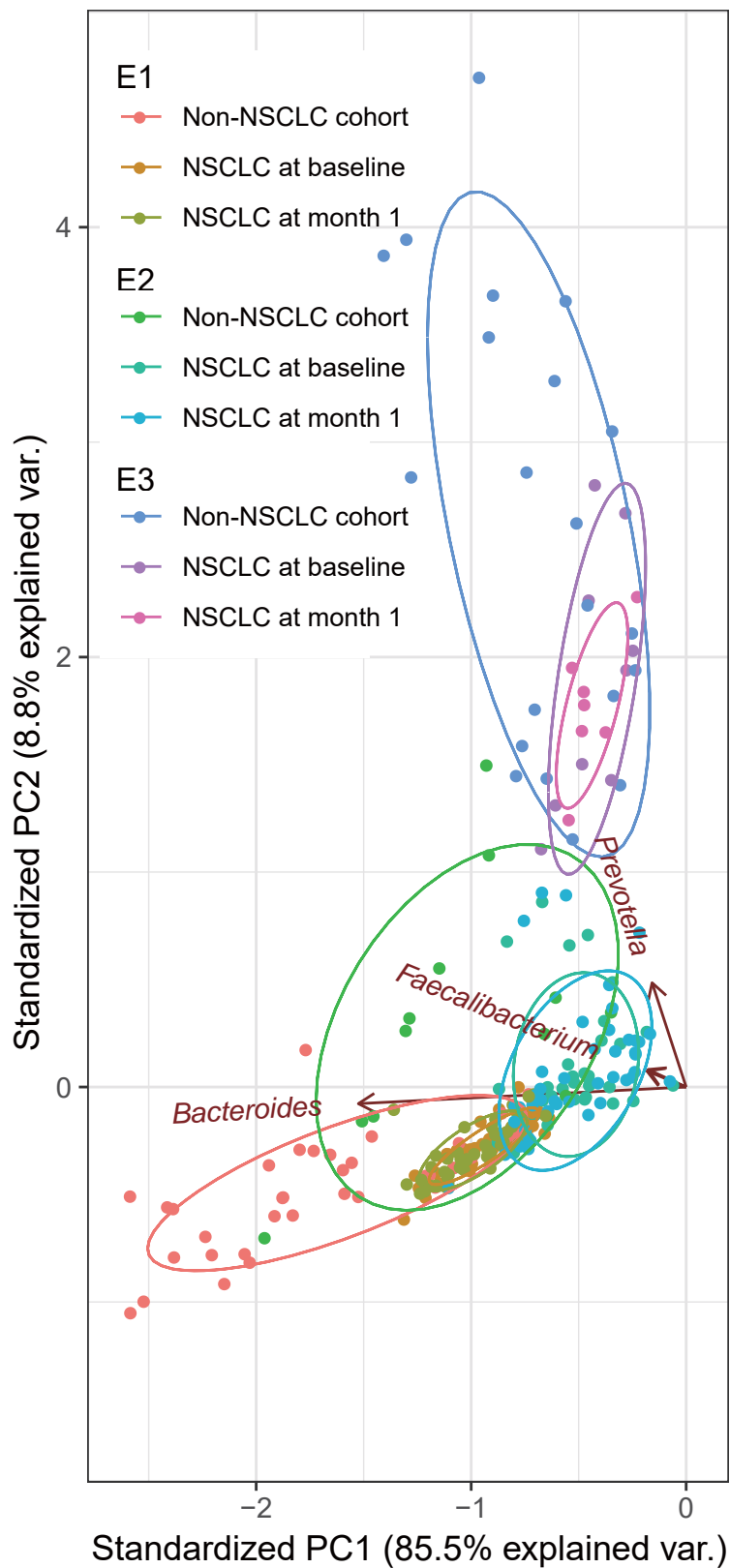

Supplement: Supplementary Figure 3 — Comparison of the gut microbiota composition at the enterotypes level between Chinese NSCLC patients and healthy controls. Samples collected at baseline (M0) and the first month (M1) after ICT initiation were selected for enterotype assessment. (A), A healthy age- and sex-matched Chinese cohort was included for background comparison. (B), Enterotype numbers were calculated at the genus level using the DMM model11. The three dominating genera for each enterotype are shown, in which type 1 (E1) was mainly driven by Bacteroides, type 2 (E2) by Faecalibacterium, and type 3 (E3) by Prevotella. (C), PCA plot showing the distribution of samples belonging to the three enterotypes. [file Image_3.pdf]

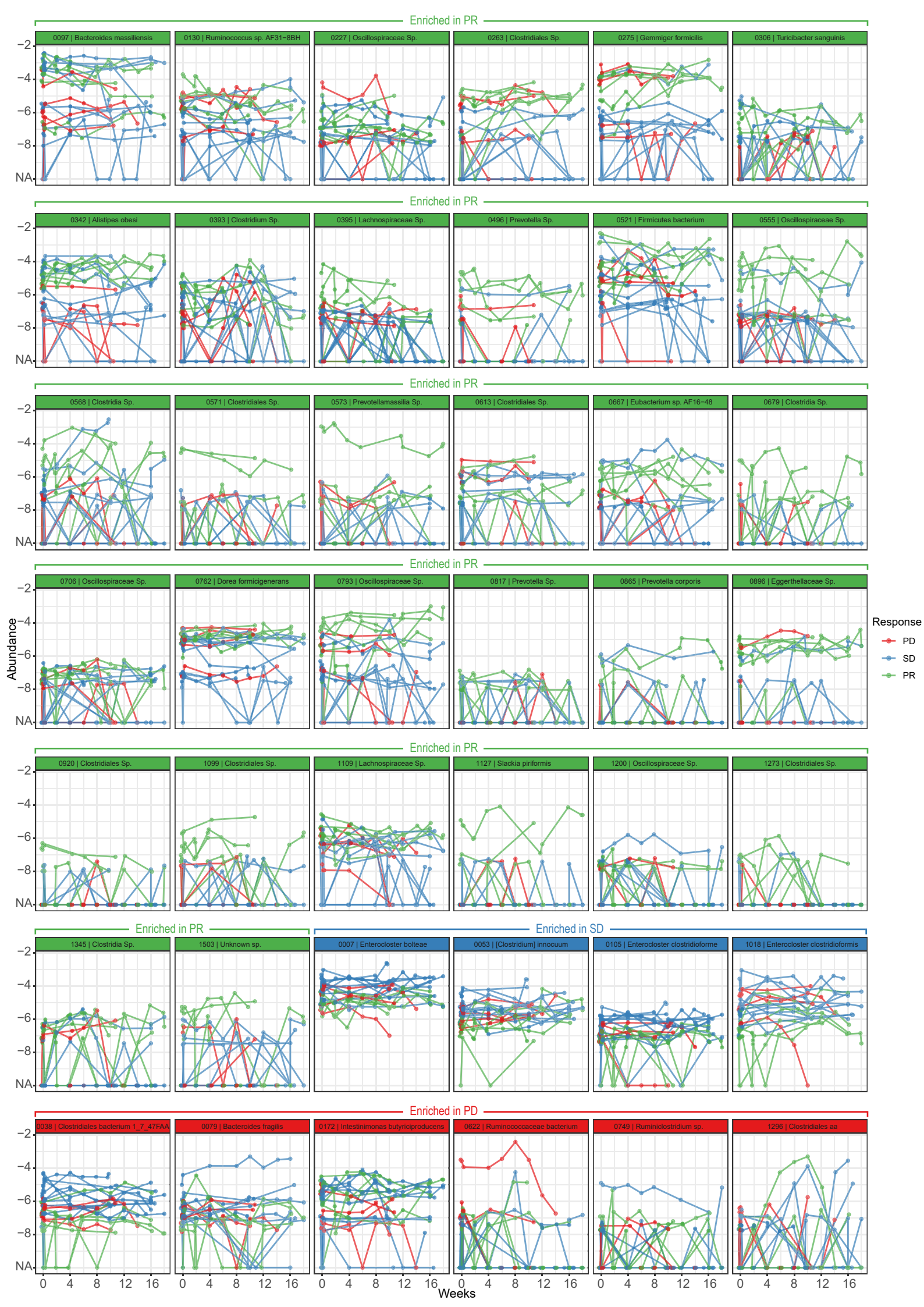

Supplement: Supplementary Figure 4 — Relative abundance of Individual MGSs at each time point. MGSs that differed in abundance between response groups are visualized. To reveal longitudinal trends, patients donating M3 samples are visualized. [file Image_4.pdf]

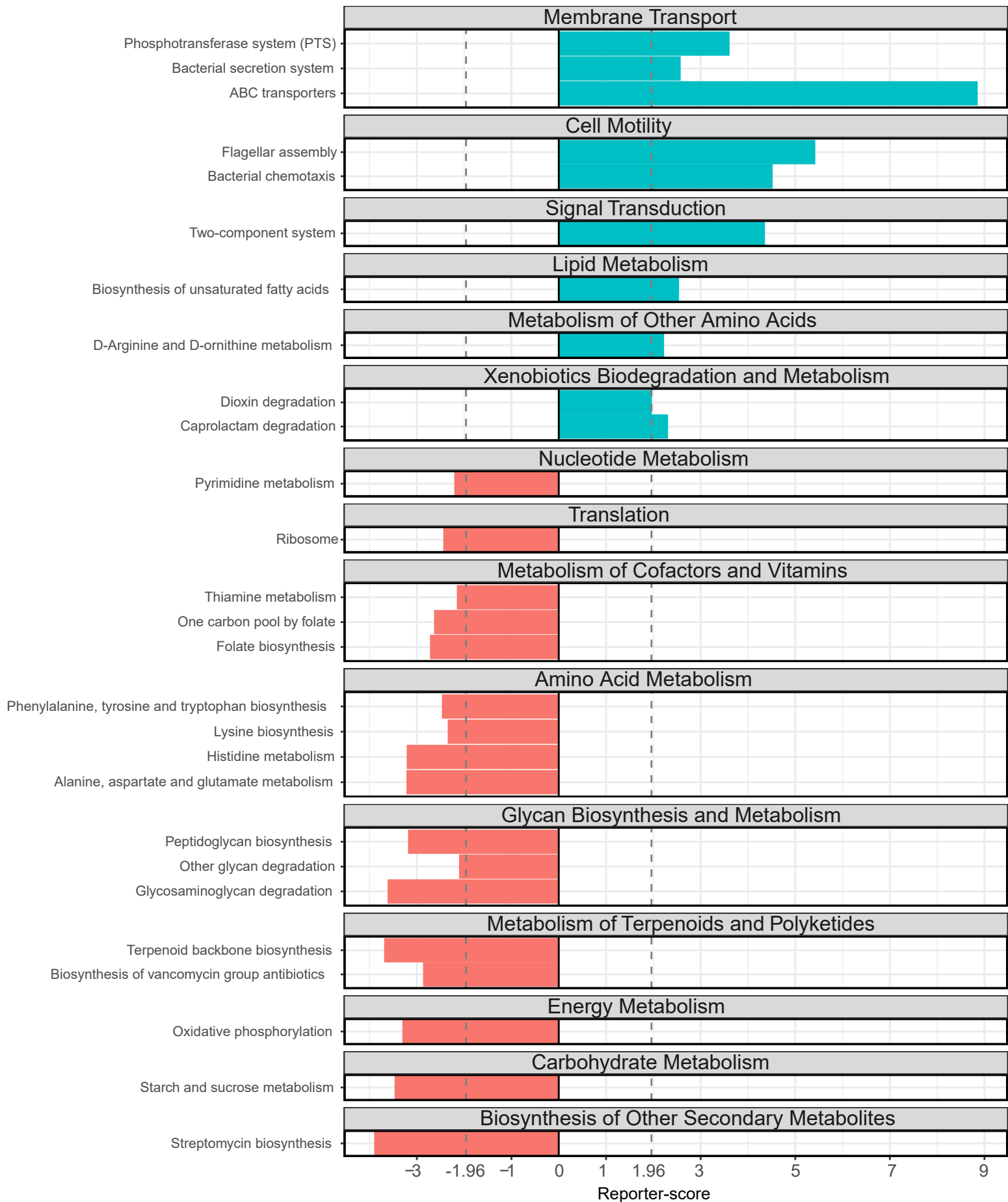

Supplement: Supplementary Figure 5 — Flagellar assembly and related pathways enriched in the PR versus the PD group. Gut microbial pathways where the reporter-score reached statistical significance are listed, displayed as the z-score calculated between the PR and PD groups. A positive value means that the pathway was enriched in the PR group, while negative values indicate an enrichment in the PD group. Pathways were derived from KEGG. [file Image_5.pdf]

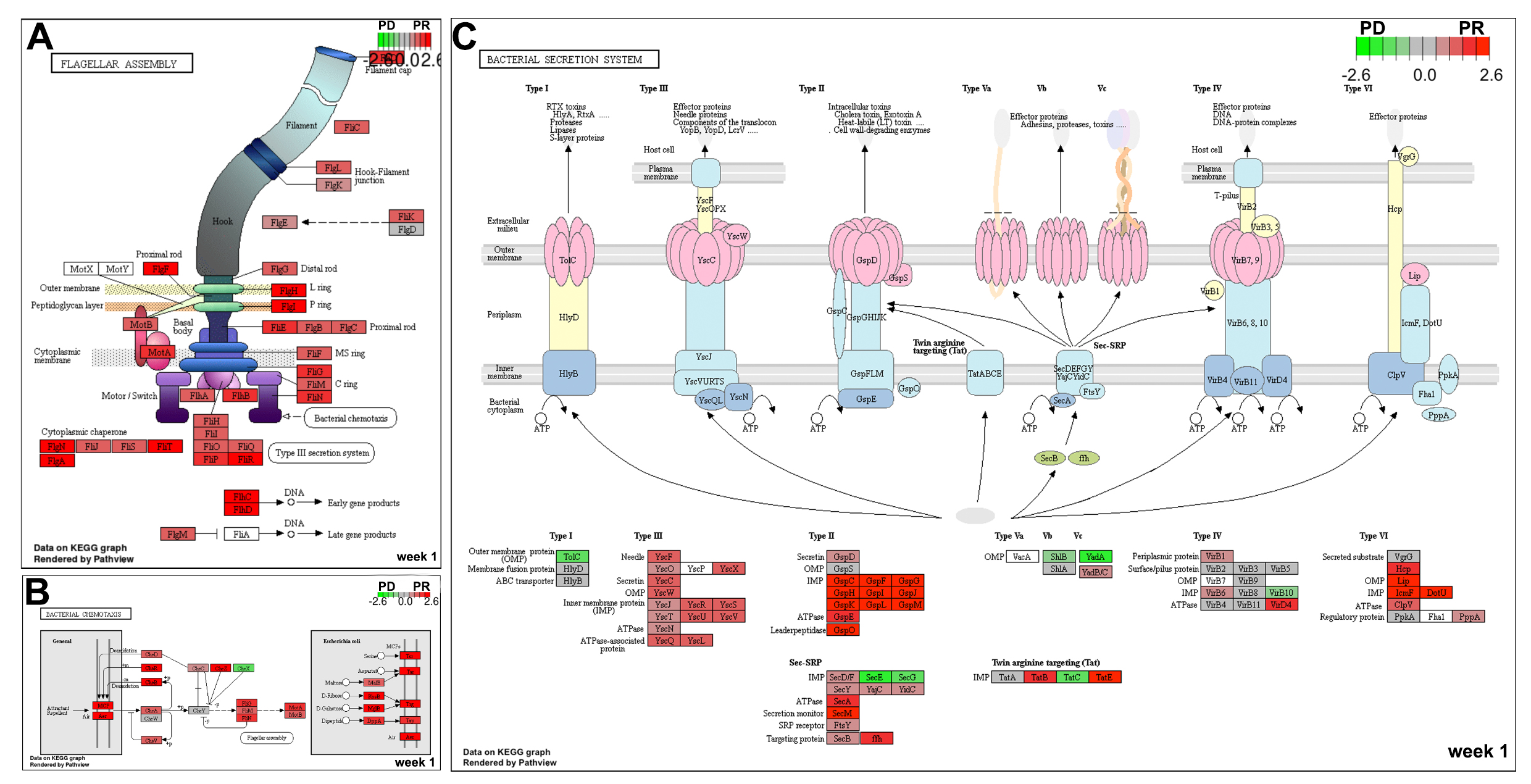

Supplement: Supplementary Figure 6 — Flagellar assembly, bacterial chemotaxis and bacterial secretion system pathway enrichment in partial responders versus disease progressors. Two KEGG pathways, flagellar assembly (A) and bacterial chemotaxis (B), both playing a part in cellular motility, contributed with the highest average reporter-score to the difference between PR and PD patients. Most of the enzymes in the flagellar assembly pathway were enriched in the PR group. Bacterial chemotaxis is also related to flagellar assembly and was similarly enriched in the PR group (C). The z-score (color) is based on PR/PD. [file Image_6.jpeg]

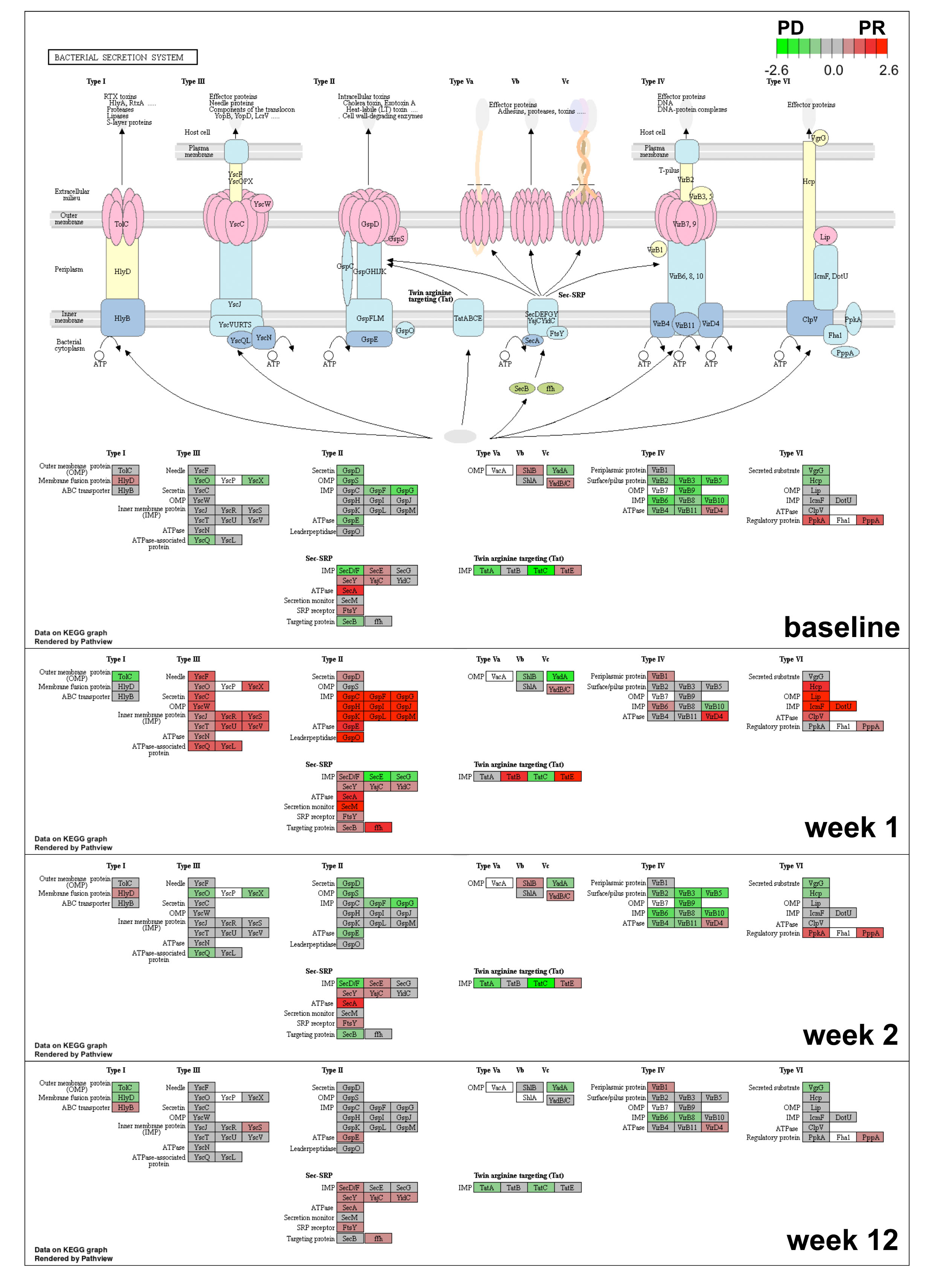

Supplement: Supplementary Figure 7 — Time dependent enrichment of KOs belonging to the bacterial secretion system pathway in the PR group. The bacterial secretion system is involved in membrane transport, especially in selected pathogens. Among six types of secretion proteins, type III, type II and type VI were enriched in the PR group during week 1 (W1). Type II is able to secrete intracellular toxins, while type II and III and type VI are both capable of secreting effector proteins into host cells. The z-score (color) is based on PR/PD. [file Image_7.jpeg]

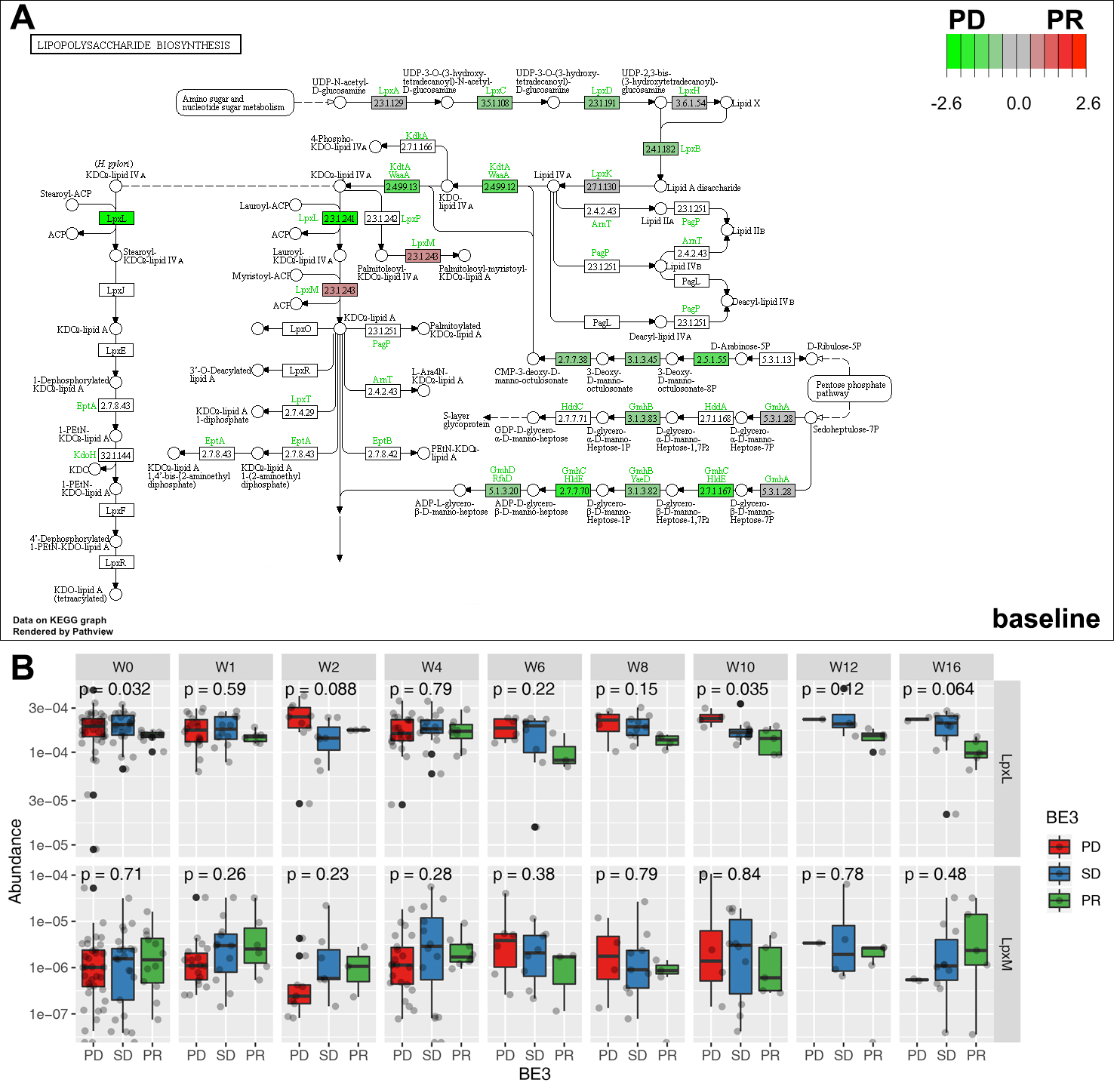

Supplement: Supplementary Figure 8 — Enriched KOs in the LPS pathway in different response groups. (A), Most enzymes in the LPS pathway were enriched in the PD group, resulting in production of penta-acylated LPS, while LpxM was enriched in the PR group, allowing for generation of hexa-acylated LPS by the LpxM-containing bacteria. The pattern in a) existed at baseline, week 1, week 2 and after 4 months of ICT. The z-score (color) is based on PR/PD. (B), Relative abundance (log10 scale) of KOs annotated to LpxM and LpxL at each time point. [file Image_8.jpeg]

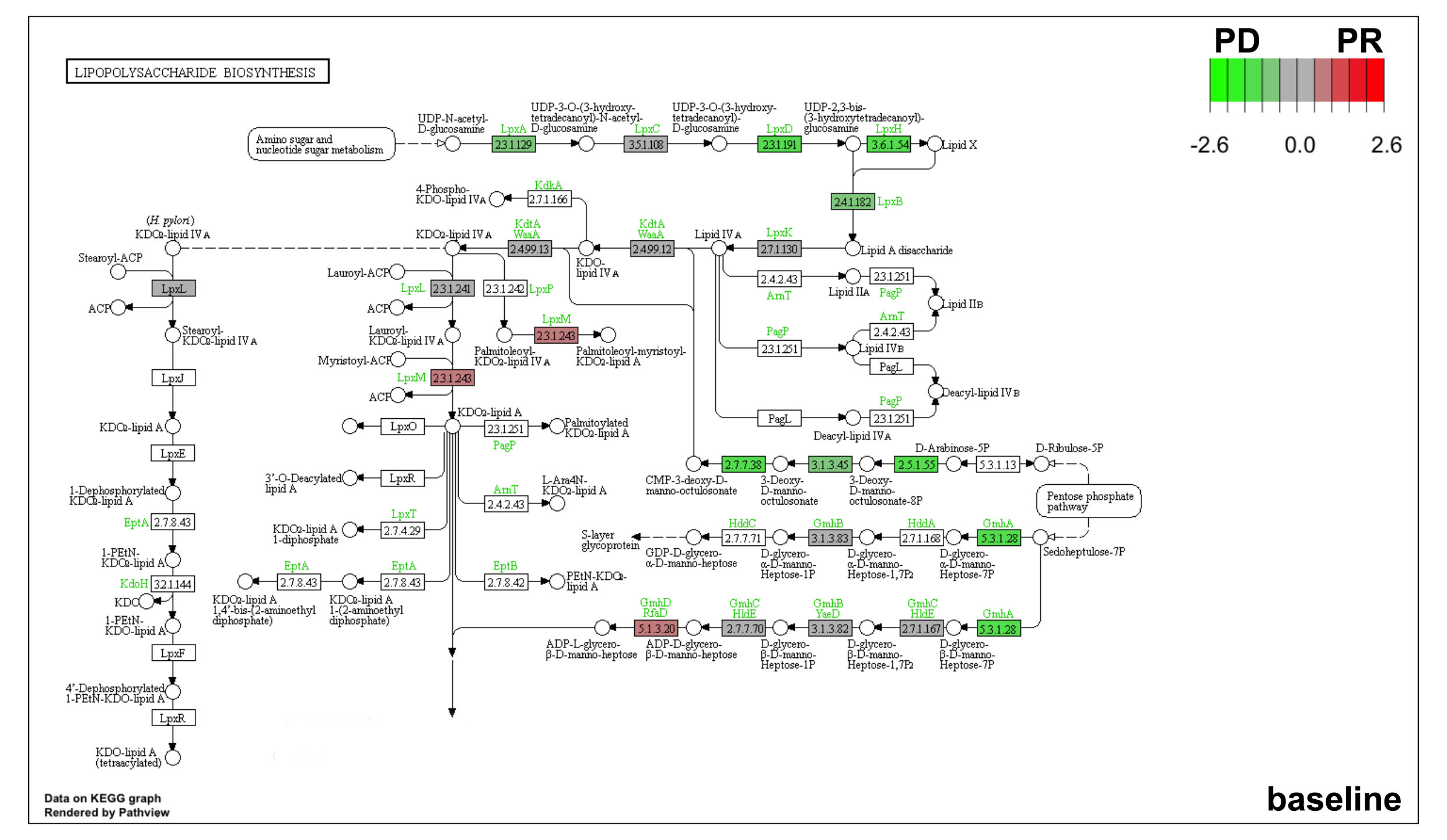

Supplement: Supplementary Figure 9 — Enriched KOs in the LPS pathway in the different response groups of a French NSCLC cohort. The reporter-score for each of the LPS pathway enzymes were enriched in a NSCLC French cohort as well as in the Chinese cohort. [file Image_9.jpeg]

**A**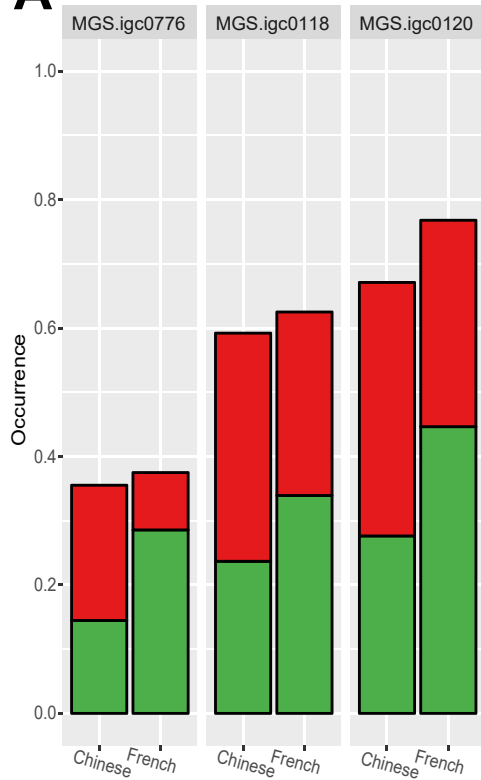**B**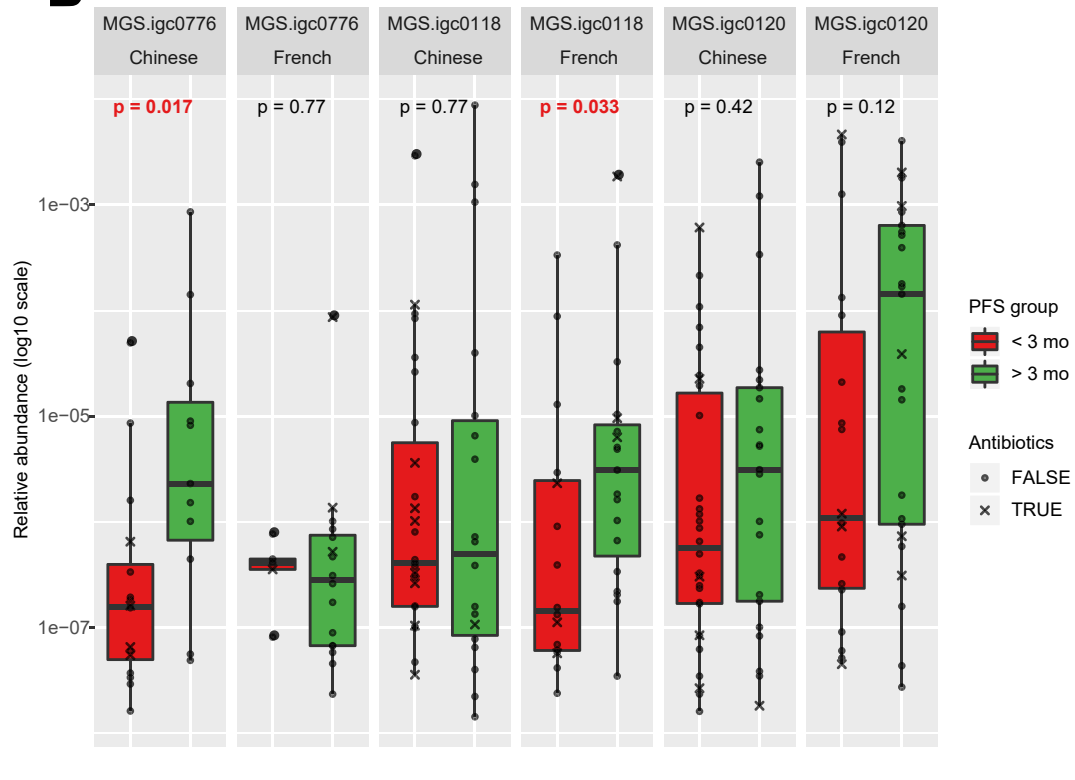

Supplement: Supplementary Figure 10 — Occurrence and abundances of Akkermansia muciniphila in a French and Chinese NSCLC cohort. Akkermansia muciniphila MGSs were identified in the two cohorts and compared. (A), Occurrence of three A. muciniphila MGSs across Rs (green) and NRs (red). (B), Abundance of the three A. muciniphila MGSs across Rs and NRs in Chinese and French NSCLC patients. [file Image_10.pdf]

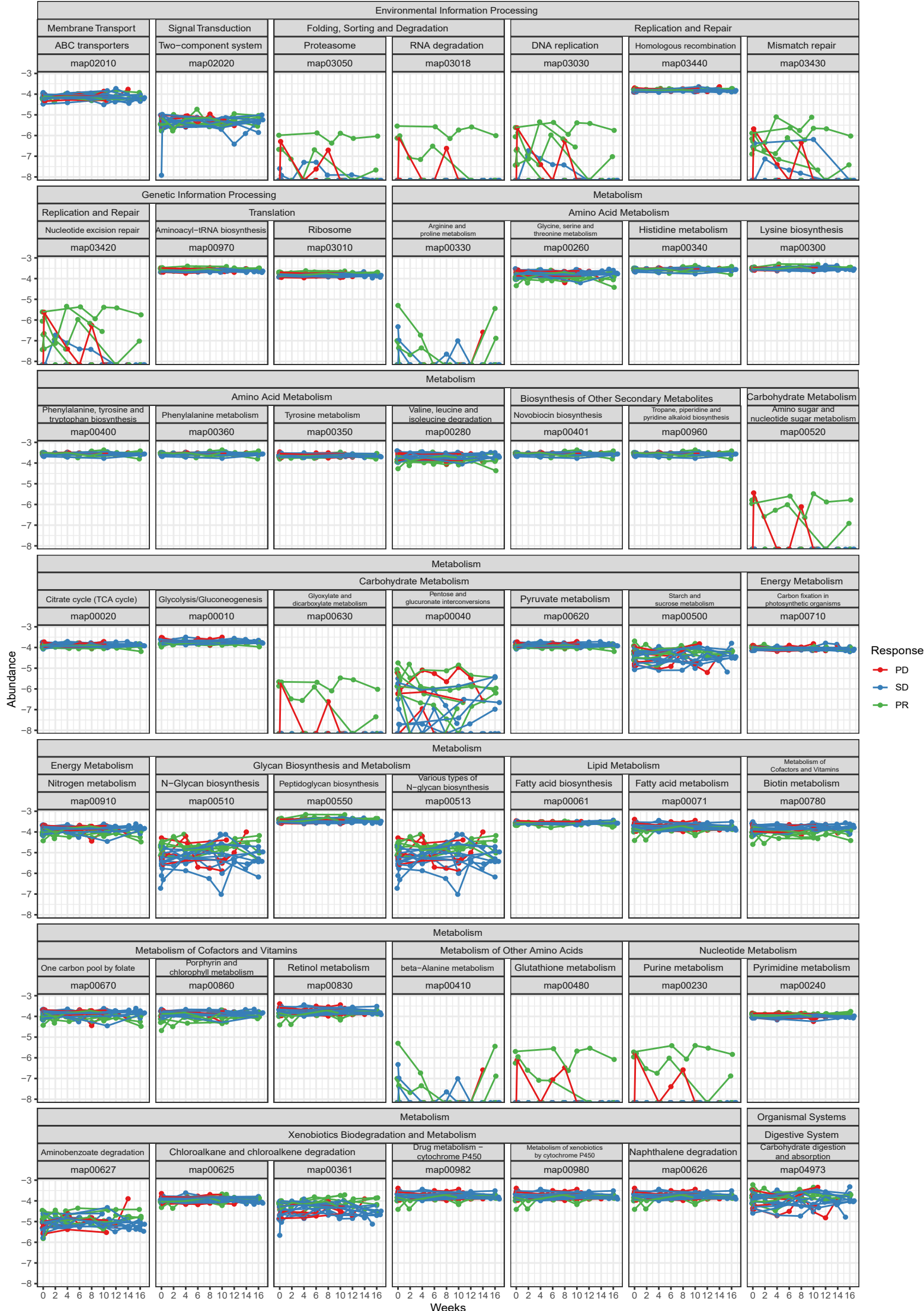

Supplement: Supplementary Figure 11 — Relative abundance of individual pathways at each time point. KOs that differed in relative abundance between the response groups were averaged to reveal longitudinal trends. Only patients donating M3 samples are visualized. [file Image_11.pdf]
