# Supplementary material for: Distinct Functional Metagenomic Markers Predict the Responsiveness to Anti-PD-1 Therapy in Chinese Non-Small Cell Lung Cancer Patients
Source: Front Oncol. 2022 Apr 21;12:837525. doi: 10.3389/fonc.2022.837525 (PMC9069064; doi:10.3389/fonc.2022.837525)
Supplement: Supplementary Figure 2 — Fecal sample collection scheme. Samples for the first week were collected within 3 days after the first ICT treatment. For each patient, the sample collected closest, but prior, to the next ICT treatment was selected as the representative sample. [file Image_2.pdf]

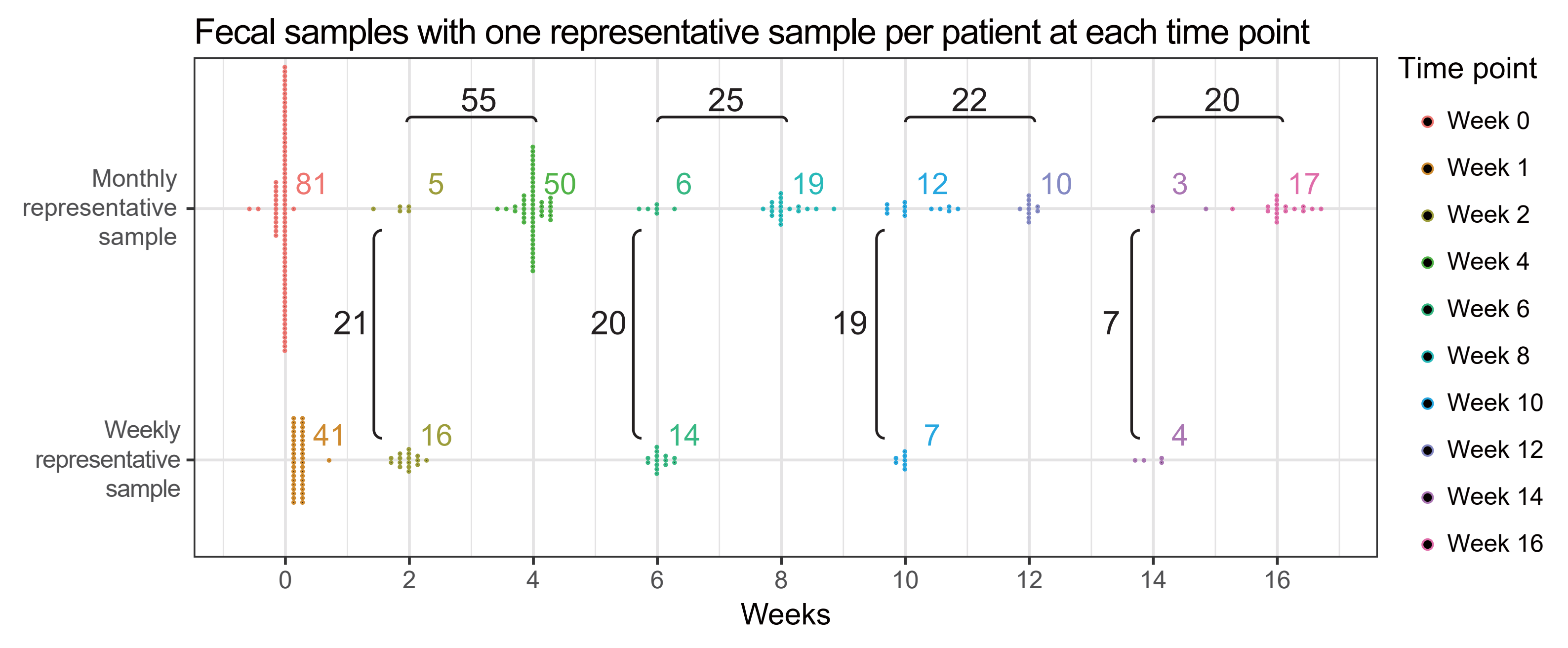

Fecal samples with one representative sample per patient at each time point
Monthly representative sample
Weekly representative sample
81
5
50
6
19
12
10
3
17
55
25
22
20
21
20
19
7
41
16
14
7
4
0
2
4
6
8
10
12
14
16
Weeks
Time point
Week 0
Week 1
Week 2
Week 4
Week 6
Week 8
Week 10
Week 12
Week 14
Week 16
